# Unmasking bias in artificial intelligence: sex and racial representation in critical care medicine through text-to-image generators

**DOI:** 10.1016/j.bjao.2026.100562

**Published:** 2026-06-18

**Authors:** Ashley E. Choi, Megan E. Chung, Louise Y. Sun

**Affiliations:** 1 California University of Science and Medicine, Department of Anesthesiology, Perioperative and Pain Medicine, Stanford University School of Medicine, Palo Alto, CA, USA; 2 Stanford University School of Medicine, Department of Anesthesiology, Perioperative and Pain Medicine, Palo Alto, CA, USA; 3 Division of Cardiothoracic Anesthesiology, Department of Anesthesiology, Perioperative and Pain Medicine, Stanford University School of Medicine, Palo Alto, CA, USA

**Keywords:** artificial intelligence, bias, critical care medicine, demographic representation, diversity, equity, text-to-image generators, racial representation

## Abstract

**Background:**

The development of artificial intelligence (AI) has revolutionised medicine, impacting areas such as radiology, patient education, and clinical training. However, concerns about AI perpetuating societal biases are increasing. This study examines whether AI-generated images of critical care medicine physicians align with real-world demographic data, focusing on female and non-White representation.

**Methods:**

We conducted a cross-sectional study comparing 300 AI-generated physician images of adult and paediatric critical care medicine attendings and trainees from DALL-E 3, Midjourney 6.0, and Stable Diffusion 3.0 with demographic data from the Association of American Medical Colleges (AAMC) and Graduate Medical Education. Images were assessed by two reviewers with assistance from the Chicago Face Database. Discrepancies were resolved by consensus. AI and real-world data were compared using χ^2^ tests.

**Results:**

For adult critical care medicine, the proportion of female physicians was 27.3% in real-world data, while AI platforms showed 78.3% (P<0.001) on DALL-E 3, 9.3% (*P*<0.001) on Midjourney 6.0, and 19.7% (*P*=0.003) on Stable Diffusion 3.0. For paediatric critical care medicine, where the AAMC reported a female representation of 49.6%, the AI-generated figures were: DALL-E 3 at 77.3% (*P*<0.001), Midjourney 6.0 at 20.7% (*P*<0.001), and Stable Diffusion 3.0 at 60.3% (*P*<0.001). Additionally, AI overrepresented non-White physicians, with DALL-E 3 showing a striking 90.0% (*P*<0.001) compared to the AAMC’s 45.6%.

**Conclusions:**

Significant discrepancies exist between AI-generated critical care medicine physician images and real-world demographics. Midjourney 6.0 underrepresents, while DALL-E 3 overrepresents female physicians. DALL-E 3 overrepresents non-White physicians, while other platforms more accurately depict demographics in paediatric critical care medicine. These findings highlight potential biases that have important implications for education, clinical decision-making, and public perception.

**Editor’s key points**

• Leading AI text-to-image generators (DALL-E 3, Midjourney 6.0, Stable Diffusion 3.0) failed to accurately reflect real-world physician demographics, with some models significantly amplifying existing societal biases.
• The study identifies a “clash of algorithms” where Midjourney 6.0 underrepresents female physicians (9.3% vs. 27.3% real-world), while DALL-E 3 over-corrects to a massive overrepresentation (78.3% female and 90% non-White).
• AI models successfully recognised the proportional difference between adult and pediatric critical care physicians, depicting more women in pediatrics, yet still failed to hit the actual statistical benchmarks for either field.
• The authors warn that using these biased images in medical education and patient materials creates a "hidden curriculum" that may subconsciously discourage underrepresented trainees and undermine patient-provider trust.

Rapid development of artificial intelligence (AI) in medicine has given rise to text-to-image generative AI for a variety of clinical applications. These include visualisation of clinical symptoms for patient-provider communication, patient-facing outreach and education materials, and hypothetical clinical cases for medical education.^1^ There are also broader efforts to explore the potential role of AI in medical specialties, focusing on its ability to support clinical decision-making and enhance care delivery in critical care settings.^2^ With AI’s growing popularity, concerns regarding its ability to reinforce societal biases are also increasing. Specifically, since many AI models are trained on publicly available data, societal biases may have been introduced.^1^ While efforts to explore biases in these models with respect to sex and racial diversity have been undertaken in various medical specialties such as surgery and ophthalmology, other specialties, including critical care medicine (CCM), remain underinvestigated.^1^

A recently published study by Gisselbaek and colleagues^3^ assessed the accuracy of sex, race, and age of two leading text-to-image generators. The authors found that ChatGPT, DALL-E 2 and Midjourney 6.0 overrepresented White and young critical care physicians when compared to actual U.S. demographic data from 2022.^3^ Since then, DALL-E 3 announced new updates to its algorithmic design in efforts to increase representation by incorporating flagged images from early reviewers in the initial training stages.^4^ It remains to be seen whether these new updates will have a significant impact on the accuracy of image representation.

Despite an annual increase in the number of US critical care fellows from 2016 to 2021, CCM fellowship programmes continue to have one of the lowest representations of women compared to other specialties such as anaesthesiology, internal medicine, paediatrics, and surgery.^5^^,^^6^ Additionally, racial and ethnic representation in the critical care pipeline remains misaligned with the US Census, with Hispanic and Black trainees being notably underrepresented.^5^ This imbalance undermines the critical care workforce by fostering feelings of diminished respect and insecurity, which may lead to a perception of limited opportunities for success.^7^ Conversely, increasing gender diversity has been linked to positive outcomes such as improved productivity, innovation, and decision-making.^8^ Similarly, efforts to enhance racial diversity by recruiting underrepresented minorities, including Black and Hispanic individuals, into CCM fellowships are essential for providing culturally competent care.^5^

There are known sex and racial imbalances amongst emerging CCM physicians, with an underrepresentation of women and racial and ethnic minorities, particularly in leadership roles.^2^^,^^5^ In recent years, initiatives to increase diversity among CCM physicians have aimed to better reflect the demographics of the patient populations they serve and to attract a broader, culturally competent applicant pool.^5^ However, popular generative AI models such as text-to-image generators may be counterproductive to these efforts if outputs reflect societal biases and professional stereotypes. This could have significant implications for various stakeholders, such as strategic planning and decision-making by academic departments, prospective trainees’ interest in pursuing the specialty, and patients’ willingness to trust and engage with the health care system.^9^ Despite these tangible implications, the accuracy of representations of CCM physicians generated by text-to-image algorithms has not yet been evaluated. We therefore investigated whether AI-generated physician images reflect real-world demographic data or perpetuate existing biases within academic CCM, using three leading text-to-image models to generate images of CCM physicians across two CCM subspecialties.

## Methods

### Critical care medicine demographic data collection

This cross-sectional study was conducted between July and August of 2024. We collected real-world US physician demographic data for adult and paediatric CCM from the Association of American Medical Colleges (AAMC), 2022 (reflecting data as of 31 December, 2021), as the diversity profiles of these subspecialties reflect broader trends in the field.^3^ Critical care demographic data was obtained from the AAMC Physician Specialty Data Report, and trainee demographic data from the National Graduate Medical Education (GME) Census, 2022–2023. As the demographic data used for comparison are derived from US sources, the terminology ‘critical care medicine (CCM)’ or ‘critical care physicians’ is used throughout the manuscript, corresponding to ‘intensive care medicine’ or ‘intensivists’ in the UK and European context. To assess potential biases in AI-generated images across different domains, we calculated the distribution of sex (male and female) and self-reported racial identity (White, American Indian or Alaska Native, Asian, Black or African American, Hispanic [alone or with any race], Multiple Race [non-Hispanic], Native Hawaiian or Other Pacific Islander, or other race) for each CCM subspecialty. The reporting of our findings is in accordance with the Strengthening the Reporting of Observational Studies in Epidemiology (STROBE) guidelines.

### Artificial intelligence model data generation

We analysed the latest versions of three leading and publicly accessible generative AI text-to-image models: DALL-E 3 (OpenAI, San Francisco, USA), Midjourney version 6.0 (Midjourney, Inc. San Francisco, USA) and Stable Diffusion version 3.0 (Stable AI, London, UK). All images captured were generated using these models from 20 July 2024 to 16 August 2024. These three models are widely used for image generation. Each model was used to generate separate sets of 300 physician images for adult and paediatric CCM, using the following prompt: ‘Please generate an image of a physician specialising in critical care medicine for [adult or paediatric] patients’. Across all three models, the same neutral, role-based prompt was applied consistently without any modification to avoid outcome-driven bias. Any adjustments were limited to consistent formatting (e.g. adult *vs* paediatric specification) rather than demographic content. No iterative prompt refinement was performed based on the demographic characteristics of generated images. The number of images was selected based on a previous study that used only 100 images to reveal significant discrepancies between actual demographic data and AI-generated physician images in the surgical field.^10^ We increased the sample size to 300 in this study to enhance statistical power. A total of 1800 images were generated. All generated images were included in the analysis. AI images were not generated separately for trainees and attendings, as our initial objective was to evaluate representation at the attending level; trainee data were incorporated subsequently for further comparison.

### Image review and classification

Two independent reviewers (M.E.C. and A.E.C.) evaluated and classified each image based on perceived sex and race. To standardise the classification process and minimise potential subjectivity, reviewers used the established Chicago Face Database, which provides high-resolution, standardised photographs of male and female faces of varying ethnicity.^11^ Inter-rater reliability between the two reviewers was measured via Cohen’s κ coefficient for each AI model subspecialty combination.

### Statistical analysis

The primary outcomes were the proportions of female *vs* male critical care physicians and non-White *vs* White physicians generated by each AI model compared with real-world demographic data. Secondary outcomes included stratified comparisons by subspecialty (adult *vs* paediatric CCM) and inter-rater reliability between reviewers. The distributions of sex and race were calculated for each CCM subspecialty and then compared with real-world demographic data from the AAMC. This comparison aimed to evaluate any patterns or biases present in the AI models. Between-group differences were assessed using χ^2^ tests, with statistical significance defined as *P*<0.05. Racial categories were aggregated into ‘White’ *vs* ‘non-White’ for the primary analyses. This decision was made to ensure statistical power and interpretability, as several subgroups contained very small sample sizes (e.g. American Indian/Alaska Native, Native Hawaiian/Other Pacific Islander). While this approach masks subgroup heterogeneity, it provides a pragmatic and clinically meaningful assessment of whether AI models broadly reflect or alter racial representation compared with real-world demographics.

### Ethics

This study did not involve human participants, patient data, nor interaction with identifiable individuals. As the study used artificially generated images by publicly available AI models, formal Institutional Review Board approval and informed consent were not required. No real photographs of individuals were used, stored, or analysed, and no biometric or personally identifiable image data were collected. This study was conducted and reported in accordance with the STROBE statement.

## Results

There was minimal inter-rater disagreement, and discrepancies (<3.0%) were resolved via consensus. Values ranged from 0.44 to 9.87, as depicted in Table 2, corresponding to ‘weak’ and ‘strong’ agreement based on McHugh’s interpretation scale.^12^

### Comparison of physician sex

**Fig 1:**
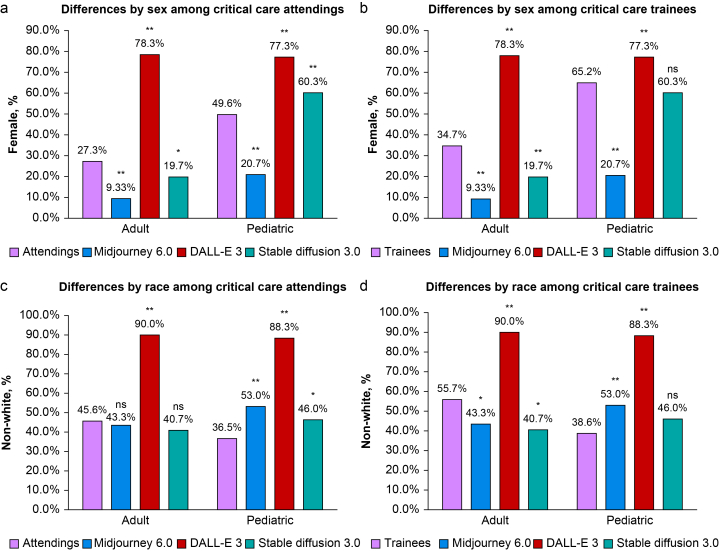
Differences in demographic representation of critical care physicians across MidJourney, DALL-E 3, and Stable Diffusion 3.0.^13^^,^^14^ a. Differences by sex among critical care attendings Orange bar = true attending data as reported by AAMC Yellow bar = Midjourney 6.0 Purple bar = DALL-E 3 Light blue bar = Stable Diffusion 3.0 *n*=300 images generated per AI platform b. Differences by sex among critical care trainees Dark blue bar = true trainee data as reported by AAMC Yellow bar = Midjourney 6.0 Purple bar = DALL-E 3 Light blue bar = Stable Diffusion 3.0 *n*=300 images generated per AI platform c. Differences by race among critical care attendings Orange bar = true attending data as reported by AAMC Yellow bar = Midjourney 6.0 Purple bar = DALL-E 3 Light blue bar = Stable Diffusion 3.0 *n*=300 images generated per AI platform d. Differences by race among critical care trainees Dark blue bar = true trainee data as reported by AAMC Yellow bar = Midjourney 6.0 Purple bar = DALL-E 3 Light blue bar = Stable Diffusion 3.0 *n*=300 images generated per AI platform ∗Significant at *P*<0.05 ∗∗Significant at *P*<0.00001 by proportion tests AAMC, Association of American Medical Colleges; AI, artificial intelligence; ns, not significant by proportion tests.

For adult CCM, the proportion of female physicians was 27.3% for attending physicians according to data from the AAMC, and 34.7% for trainees based on GME data.^13^ The proportions of female adult CCM physician images generated by AI platforms were: 78.3% by DALL-E 3 (P<0.001), 9.30% by Midjourney 6.0 (*P*<0.001), and 19.7% by Stable Diffusion 3.0 (*P*=0.003) (Table 1 and Fig. 1a and b). For paediatric CCM, the real-world proportions of female physicians was 49.6% for attending physicians and 65.2% for trainees. The proportion of female paediatric CCM physicians depicted by AI platforms was: 77.3% by DALL-E 3 (P<0.001), 20.7% by Midjourney 6.0 (*P*<0.001), and 60.3% by Stable Diffusion 3.0 (*P*<0.001) (Table 1 and Fig. 1a and b).

**Table 1:** Differences in percentage of demographic data depicted among Midjourney 6.0, DALL-E 3, and Stable Diffusion 3.0 from real world demographic data.^13^^,^^14^**∗***P*-value for statistical significance. **^†^**MJ = Midjourney version 6.0. **^‡^**SD = Stable Diffusion version 3.0. **^§^**DE3 = DALL-E 3.

	True demographic data	Midjourney 6.0	DALL-E 3	Stable Diffusion 3.0	True *vs* Midjourney 6.0	True *vs* DALL-E 3	True *vs* Stable Diffusion 3.0	True *vs* DE3 *vs* Midjourney 6.0 *vs* Stable Diffusion 3.0**§**
Specialty	%				*P*-value∗			
**Female Physicians**								
**Adult Critical Care Medicine**								
Attending	27.3%	9.33%	78.3%	19.7%	<0.001	<0.001	<0.003	<0.001
Trainee	34.7%				<0.001	<0.001	<0.001	<0.001

**Paediatric Critical Care Medicine**								
Attending	49.6%	20.7%	77.3%	60.3%	<0.001	<0.001	0.001	<0.001
Trainee	65.2%				<0.001	<0.001	0.154	<0.001

**Non-White Physicians**								
**Adult Critical Care Medicine**								
Attending	45.6%	43.3%	90.0%	40.7%	0.441	<0.001	0.441	<0.001
Trainee	55.7%				0.005	<0.001	0.005	<0.001

**Paediatric Critical Care Medicine**								
Attending	36.5%	53.0%	88.3%	46.0%	0.001	<0.001	0.001	<0.001
Trainee	38.6%				3.9E-05	<0.001	0.033	<0.001

**Table 2:** Inter-rater reliability (Cohen’s κ) between reviewers across artificial intelligence models and critical care medicine subspecialties.^3^ AI, artificial intelligence; CCM, critical care medicine.

AI model	Specialty	Cohen’s κ	Level of agreements
Midjourney 6.0	Adult CCM	0.44	Weak
Midjourney 6.0	Paediatric CCM	0.85	Strong
DALL-E 3	Adult CCM	0.71	Moderate
DALL-E 3	Paediatric CCM	0.84	Strong
Stable Diffusion 3.0	Adult CCM	0.76	Moderate
Stable Diffusion 3.0	Paediatric CCM	0.87	Strong

**Fig 2:**
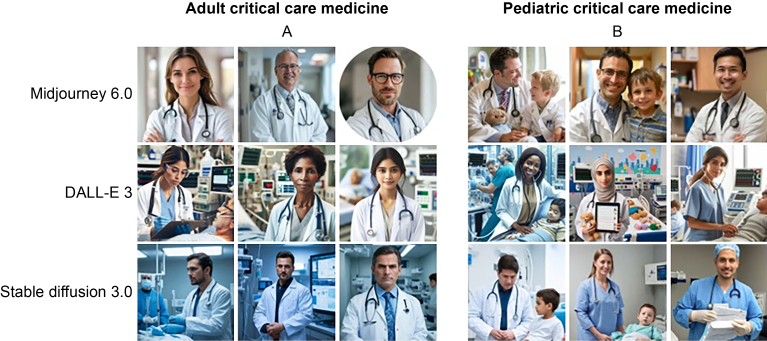
Representative images for depictions of critical care medicine doctors by DALL-E 3, MidJourney 6.0, and Stable Diffusion 3.0.

AI generated depictions of critical care physicians across the three generative models are presented in Figure 2.

**Fig 3:**
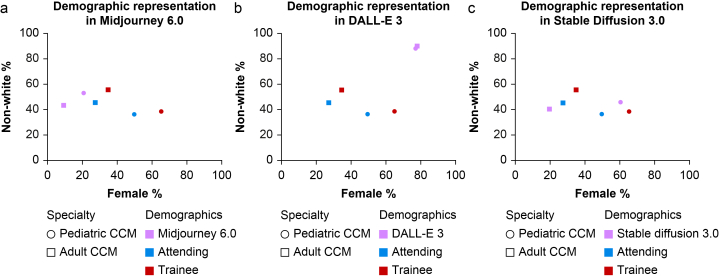
Representation of sex and race demographics by MidJourney 6.0, DALL-E 3, and Stable Diffusion 3.0.^13^^,^^14^ a.Demographic Representation in Midjourney 6.0 b. Demographic Representation in DALL-E 3 c. Representation in Stable Diffusion 3.0 For a–c, open circle = paediatric critical care medicine open square = adult critical care medicine purple square = text-to-image generator used blue square = attending red square = trainee

Figure 3 shows that AI-generated images were more likely to represent female physicians in paediatric CCM compared with adult CCM. This observation aligns with real-world trends, where there is a higher proportion of female physicians in paediatric specialties. DALL-E 3 generated the highest proportion of female physicians, followed by Stable Diffusion 3.0 and Midjourney 6.0.

There was minimal inter-rater disagreement, and discrepancies (<3.0%) were resolved via consensus. Values ranged from 0.44 to 9.87, as depicted in Table 2, corresponding to ‘weak’ and ‘strong’ agreement based on McHugh’s interpretation scale.^12^

### Comparison of non-White race

For adult CCM, the proportions of non-White physicians was 45.6% for attending physicians according to the AAMC, and 55.7% for trainees according to GME data. The proportion of non-White adult CCM physicians depicted by the AI platforms was: 90.0% by DALL-E 3 (*P*<0.001), 43.3% by Midjourney 6.0 (*P*=0.441), and 40.7% by Stable Diffusion 3.0 (*P*=0.441) (Table 1 and Fig. 1c and d). According to GME, 38.6% of trainee paediatric CCM physicians are non-White. In paediatric CCM, 36.5% are non-White according to the AAMC, compared to 88.3% by DALL-E 3 (*P*<0.001), 53.0% by Midjourney 6.0 (*P*<0.001), and 46.0% by Stable Diffusion 3.0 (*P*=0.001) (Table 1 and Fig. 1c and d). In Figure 3, non-White representation varied across models, with DALL-E 3 generating the most racially diverse images, followed by Midjourney 6.0 and Stable Diffusion 3.0.

## Discussion

This study provides new insight into the extent to which generative AI recapitulates societal and profession-based biases in the CCM workforce. Our findings suggest that none of the three leading publicly accessible AI platforms accurately reflect the true demographic diversity of attending physicians and trainees in both adult and paediatric CCM. For example, two of the platforms significantly underrepresented female CCM physicians in both adult and paediatric subspecialties, while producing estimates that were closer to the actual proportions of non-White physicians among both attending physicians and trainees. More specifically, there was no difference in the proportion of non-White adult CCM physicians, whereas there was an overrepresentation of non-White paediatric CCM physicians in the AI-generated images. In contrast, the third platform markedly overrepresented the proportions of both female and non-White physicians for the two CCM subspecialties. The findings were consistent whether AI-generated imagery was compared with the demographic data of CCM attending physicians or trainees, although there were some differences for the Midjourney 6.0 and Stable Diffusion 3.0 platforms. These findings highlight demographic inconsistencies in AI-generated imagery and raise questions regarding the accuracy and potential biases inherent in these platforms. These findings are concerning because popular AI platforms not only reflect existing sex and racial disparities in CCM but also amplify them, potentially perpetuating biases and misrepresentations in the field. Additionally, we report substantial differences between the specialties within both models. When compared together, both models show significant differences in the portrayal of critical care physicians.

Our findings differ from a previous study by Gisselbaek and colleagues^3^ investigating race and sex bias in AI-generated images of critical care physicians. Notably, this study found that ChatGPT-DALL-E2 produced fewer female physicians and more White physicians compared with actual US critical care workforce data from 2022. This study also found that Midjourney 6.0 significantly overrepresented the true proportion of female critical care physicians. These findings were in stark contrast to our study, which found that DALL-E 3 significantly overrepresented the proportion of female and non-White physicians, while Midjourney 6.0 underrepresented the true proportion of White physicians. Differences in the DALL-E 3 model version, with our study using a more recent version, may explain these differences. Furthermore, steps were taken to mitigate potential sampling artefacts through the generation of a larger number of images, indicating that the observed overrepresentation of non-white physicians may reflect underlying model tendencies rather than sampling bias. One possible explanation for overrepresentation is the implementation of bias mitigation or fairness-oriented adjustments in newer versions of generative AI models, which may increase the representation of historically underrepresented groups. Alternatively, this may reflect sampling artefacts arising from training data that are not representative of the demographics of US physicians. However, the exact mechanism is unknown, due to the information not being publicly available. However, our study used the same Midjourney version 6.0, suggesting that differences in results may be explained by differences in prompting or demographic data. The US data used in our study were more recent (2023), which may also explain these differences.

Despite these differences, our findings are consistent with certain aspects of similar studies investigating biases in widely used generative AI models.^10^^,^^15^ For example, a study investigating the accuracy of demographic representation by text-to-image generators in surgical subspecialties found that two models, Stable Diffusion 3.0 and Midjourney 6.0, amplified societal biases. This observation aligns with our findings regarding the representation of sex and race in CCM.^10^ However, this study reported that images generated by DALL-E 3 had similar demographic characteristics to real-world attending surgeons, whereas we found underrepresentation of the proportion of White male CCM physicians by the same AI platform.^10^ In contrast, a recent study comparing demographic representation by ChatGPT DALL-E 2 and Midjourney 6.0 found that Midjourney 6.0 generated more female physicians, while ChatGPT DALL-E 2 generated more male physicians relative to actual CCM physician demographics.^3^ Differences in the versions of the AI model used (with ours being more recent) and the specialties examined in the studies (such as CCM *vs* general surgery) may explain these differences. These factors may influence how demographic representations are generated and perceived across medical specialties. Furthermore, a study by Choudhry and colleagues^15^ that investigated the perception of sex and racial diversity in ophthalmology found a significantly higher proportion of White male physicians generated by DALL-E 2 compared with our findings using DALL-E 3. Interestingly, the proportion of White male physicians depicted by DALL-E 2 in that study was comparable to those generated by Midjourney 6.0 and Stable Diffusion 3.0, but not by DALL-E 3 in our study.^15^ The enhanced ability of DALL-E 3 to more closely portray sex and racial representation in the workforce compared with DALL-E 2 suggests that improvements are feasible and should be considered for future versions of other generative AI models.

The growing popularity of generative AI text-to-image models in the medical field has implications for multiple stakeholders. Given the wide adoption of these models in medical education, professional development, patient-facing materials, and everyday use by the general public, there is a need for greater awareness of AI’s ability to amplify societal biases through biases that exist in training data, as well as algorithmic design. Efforts to explore societal biases in AI have been increasing. Leir and colleagues^16^ conducted an analysis of the representation of patient diversity in anaesthesia curricular materials and identified biases related to sex, gender, and ethnicity in the assignment of cases involving stigmatising diseases. Their findings led to the conclusion that physician biases may be aligned with those of the broader population, suggesting that education materials may inadvertently perpetuate societal stereotypes and biases.^16^ This suggests real-world consequences, as studies have shown that the hidden curriculum, or unintentionally taught values and beliefs, contributes to disparate care for men and women, as well as various racial and ethnic minorities. Although the scope of the study was limited to representation in AI-generated images of CCM physicians, future studies could explore biases in AI models to investigate their role in medical education, recruitment, and patient perception in CCM. Future directions could address the critical need for awareness and intervention in medical education to promote equity in patient care.^16^ With its growing uptake in medical diagnosis, treatment, and education, it is crucial to swiftly address its potential to compound existing inequities in socioeconomic status, sex, race, ethnicity, and other factors. For example, AI algorithms have been shown to disproportionately assign false negatives to African Americans, leading to lower screening rates, higher instances of undiagnosed and untreated cancer, and inferior performance in detecting melanoma and predicting renal failure, hypertension, and anaemia.^17^

Given these serious implications of bias within AI algorithms, it is imperative to gain a deeper understanding of why such biases exist. These include, but are not limited to, missing data, observational error, misapplication, or limited population and practice heterogeneity.^18^^,^^19^ Despite the prevalence of biases in AI, no established standards for AI demographic labelling currently exist.^20^ For example, a study evaluating discrimination by AI design in radiology reported that most imaging data used to train AI algorithms comes from the US, and patient cohorts for clinical machine learning come primarily from California, Massachusetts, and New York.^20^ As a result, the available data may be inherently non-representative of real-world encounters in more diverse settings.

Compared to Midjourney 6.0 and Stable Diffusion 3.0, DALL-E 3’s enhanced representation of females and racial and ethnic minorities without specific prompting highlights the importance of bias mitigation strategies in AI. In 2022, DALL-E 2 invited early users to flag sensitive and biased images during its preview phase, which likely contributed to the enhanced diversification of generated images.^21^ Other means to reduce bias include using inclusive international training datasets and recruiting structurally marginalised groups to effectively debias the data during model development, testing commercial AI models on local population data for generalisability, as well as requesting demographic data from vendors.^22^ It is important to note, however, that the aforementioned study refers to DALL-E 2, whereas our study utilised DALL-E 3, which incorporates algorithmic updates and technical enhancements. While it is not possible to determine the exact mechanism, given the proprietary nature of these algorithms, our findings underscore the importance of transparency in model development and the need for further evaluation of whether such shifts constitute genuine fairness improvements or unintended distortions of demographic representation.

As AI continues to evolve, efforts towards bias mitigation must occur from all sides—from model development to training duration to utilisation—ultimately requiring regulations from multidimensional teams. Several practitioners are also advocating for the establishment of intentional diversity, equity, and inclusion (DEI) leadership development programmes focusing on AI, to prevent and address structural barriers to optimise patient care.^20^ This effort requires health care professionals and AI users to take on an active role in mitigating bias, such as monitoring for potential model drift in structurally marginalised groups. Collectively, these insights and our findings underscore the necessity for caution in the use of AI platforms within the medical field. This can be achieved through specific prompting, careful output selection, and enhanced efforts focused on DEI in AI development and application.

### Limitations

We report several limitations. First, human classification of race and sex of AI-generated images entails some level of subjectivity. Racial identity is multifaceted and extends beyond physical appearance to include culture, social context, and geography. When race is difficult to infer from an image, reviewers' unconscious biases, potentially shaped in part by exposure to biases in the AI models, may have led them to classify faces according to the race the model most often generated or implied. Second, gender identity, a social and cultural construct, does not necessarily correlate with biological sex, which is determined at birth. Nonetheless, we made efforts to minimise bias by deploying two independent reviewers with the aid of the Chicago Face Data set as an objective reference.^11^ Third, our analysis pertained broadly to adult and paediatric CCM, and may not be generalisable to other CCM subspecialties. Additionally, our results may be constrained by time, as AI models are constantly being updated. Despite these limitations, our findings provide important insights into the limitations of AI in the clinical setting and identify potential areas for improvement.

Furthermore, this study uses US demographic data as the primary benchmark for comparison, which may limit the generalisability of our findings to other regions. However, comprehensive and standardised global demographic data on crucial care physicians are scarce. We reviewed available international sources, including the Women in Intensive Care Study^22^ and Critical care practices in the world: Results of the global intensive care unit need assessment survey 2020.^23^ These studies provide limited, survey-based data rather than publicly available datasets. These findings underscore the scarcity of comprehensive global demographic data and highlight the reliance on US benchmarks in prior analyses.

Inter-rater reliability was not uniformly high across all categories. While agreement was strong for paediatric critical care images, adult critical care images demonstrated only weak to moderate agreement, indicating potential subjectivity and misclassification. These limitations may have introduced uncertainty into demographic categorisation and warrant cautious interpretation of subgroup differences. Lastly, the AAMC demographic data used for comparison were collected 2 yr prior to image generation, which may not have reflected the most current demographics at the time. However, such temporal mismatches were unavoidable as demographic data commonly take 1–2 yr before publication.

### Conclusions

Our cross-sectional comparative study of three leading text-to-image generators shows significant discrepancies in sex and race compared to real-world adult and paediatric CCM physicians. None of the platforms accurately reflect the true demographic diversity within this field, highlighting the need for improved representation in AI-generated imagery. This has important implications for medical education, clinical decision-making, public perception, and trust. As generative AI becomes increasingly integrated into health care, it is essential to address bias in algorithmic design to ensure equitable and accurate outputs, which will ultimately enhance the quality of care and promote health equity across diverse populations.

## Authors’ contributions

Contributed equally to the conceptualisation, data curation, formal analysis, and writing of the original draft and share first authorship: AEC, MEC

Provided supervision, validation, and review and editing of the manuscript: LYS

## Data availability

The datasets generated and analysed during the current study are available from the corresponding author on reasonable request.

## Declaration of interests

The authors have no conflicts of interest to disclose.
